# Trace and Major Element Concentrations in Cadaveric Lung Tissues from World Trade Center Health Registry Decedents and Community Controls

**DOI:** 10.3390/ijerph20206923

**Published:** 2023-10-14

**Authors:** Michael Marmor, Joyce L. Burcham, Lung-Chi Chen, Steven N. Chillrud, Jason K. Graham, Hannah T. Jordan, Mianhua Zhong, Elizabeth Halzack, James E. Cone, Yongzhao Shao

**Affiliations:** 1 Departments of Population Health and Medicine, NYU Grossman School of Medicine, New York, NY 10016, USA; 2 Department of Population Health, NYU Grossman School of Medicine, New York, NY 10016, USA; jlburcham@aol.com (J.L.B.); yongzhao.shao@nyulangone.org (Y.S.); 3 Division of Medicine, NYU Grossman School of Medicine, New York, NY 10016, USA; lung-chi.chen@nyulangone.org; 4 Lamont-Doherty Earth Observatory, Columbia University, Palisades, NY 10964, USA; chilli@ldeo.columbia.edu; 5 New York City Office of Chief Medical Examiner and Department of Forensic Medicine, NYU Grossman School of Medicine, New York, NY 10016, USA; jgraham@ocme.nyc.gov; 6 New York City Department of Health and Mental Hygiene, World Trade Center Health Registry, New York, NY 11101, USA; hjordan1@health.nyc.gov (H.T.J.); jcone@health.nyc.gov (J.E.C.); 7 Department of Medicine, NYU Grossman School of Medicine, New York, NY 10016, USA

**Keywords:** World Trade Center disaster, autopsy, lung, trace elements, exposure estimation, epidemiology, iron, molybdenum, titanium, chromium, arsenic, national technical information services (NTIS) standards

## Abstract

Studies of the health impacts of the 11 September 2001 terrorist attacks on New York City’s (NYC’s) World Trade Center (WTC) towers have been hindered by imprecise estimates of exposure. We sought to identify potential biomarkers of WTC exposure by measuring trace and major metal concentrations in lung tissues from WTC-exposed individuals and less exposed community controls. We also investigated associations of lung tissue metal concentrations with self-reported exposure and respiratory symptoms. The primary analyses contrasted post-mortem lung tissue concentrations obtained from autopsies in 2007–2011 of 76 WTC Health Registry (WTCHR) enrollees with those of 55 community controls. Community controls were frequency-matched to WTCHR decedents by age at death, calendar quarter of death, gender, race, ethnicity and education and resided at death in NYC zip codes less impacted by WTC dust and fumes. We found WTCHR decedents to have significantly higher iron (Fe) lung tissue concentrations than community controls. Secondary analyses among WTCHR decedents adjusted for sex and age showed the log(molybdenum (Mo)) concentration to be significantly associated with non-rescue/recovery exposure. Post hoc analyses suggested that individuals whose death certificates listed usual occupation or industry as the Sanitation or Police Departments had elevated lung tissue Fe concentrations. Among WTCHR decedents, exposure to the WTC dust cloud was significantly associated with elevated lung tissue concentrations of titanium (Ti), chromium (Cr) and cadmium (Cd) in non-parametric univariable analyses but not in multivariable analyses adjusted for age and smoking status. Logistic regression adjusted for age and smoking status among WTCHR decedents showed one or more respiratory symptoms to be positively associated with log (arsenic (As)), log(manganese (Mn)) and log(cobalt (Co)) concentrations, while new-onset wheezing and sinus problems were negatively associated with log(Fe) concentration. Fe concentrations among individuals with wheezing, nonetheless, exceeded those in community controls. In conclusion, these data suggest that further research may be warranted to explore the utility as biomarkers of WTC exposure of Fe in particular and, to a lesser extent, Mo, Ti, Cr and Cd in digestions of lung tissue.

## 1. Introduction

Studies of the health consequences of the WTC disaster have usually estimated exposure to WTC contaminants from self-reports [1,2,3,4]. Examples include a study that estimated firefighters’ exposure intensities from time of arrival at the WTC site and immersion in the dust cloud resulting from the collapse of the towers [4]. Another study graded firefighters’ exposure severities only from time of arrival at the disaster site, deeming high-exposed those who arrived on the morning of 11 September 2001, moderate-exposed those who arrived in the afternoon of 11 September 2001 or on 12 September 2001 and low-exposed those who arrived between 13 September 2001 and 25 July 2002 [5]. Some studies of occupationally exposed individuals have used work history to categorize exposure [6,7,8,9,10], but equivalent history is not available for members of the general public affected by the disaster. The crudeness of these exposure-estimation methods has caused imprecision in estimating relative exposure among individuals. The accurate characterization of subjects’ exposure is critical to understanding dose–response relationships, which play an important role in causal inference based on epidemiologic studies.

Airborne contaminants from the WTC event included particulate matter containing trace and major elements. If these elements were retained in tissues, they might provide useful biomarkers of exposure. WTC particles with diameters >2.5 μm were composed primarily of pulverized concrete, gypsum and synthetic vitreous fibers [11,12]. Among other elements, concrete contains iron (Fe) [13], an element also observed in WTC aerosols [14].

Two studies have investigated biopsy tissues from WTC-exposed individuals. In one study, lung biopsies of WTC responders with severe respiratory symptoms or unexplained abnormal radiographic findings found increased presence of aluminum (Al), magnesium (Mg) silicates, chrysotile asbestos (Mg_3_(Si_2_O_5_)(OH)_4_), calcium phosphate (CaPO_4_), calcium sulphate (CaSO_4_), carbon nanotubes, and glass shards containing silica (SiO_2_) and Mg [15]. In the second study, lung specimens from 12 WTC-exposed individuals with suspected interstitial lung disease (*n* = 6) or abnormal pulmonary function tests (*n* = 6) had increased presence of birefringent particles containing titanium (Ti), Al or steel (comprising Fe, chromium (Cr) and nickel (Ni)), and “diverse metals”, including zirconium (Zr), Cr, copper (Cu), zinc (Zn) and tin (Sn), compared with 9 controls [16]. A pilot study of in vivo X-ray fluorescence of the tibia for estimation of WTC exposure suggested elevations in antimony (Sb) and Ti among WTC survivors [17]. Weak signals and a small number of subjects, in part due to the reluctance of WTC survivors to consent to low-dose radiation exposure, led to the early cessation of this project. These findings suggest that particles containing trace or major elements from the WTC disaster can persist in tissues years after the event.

The New York City (NYC) Office of Chief Medical Examiner (OCME) autopsied ~5000 individuals per year during 2007–2011, comprising approximately 9% of annual NYC deaths [18]. The frequency of autopsies coupled with the large number (>70,000) of WTC-exposed individuals enrolled in the WTC Health Registry (WTCHR) suggested that stored autopsy materials from WTCHR registrants might provide an adequate sample in which to search for exposure biomarkers. We chose to analyze lung tissues, as research has suggested long-term blockage of sections of the lung by WTC particulate matter [16,19].

### Study Hypothesis and Aims

We thus investigated the hypothesis that cadaveric lung tissue obtained at autopsies of WTC-exposed individuals would contain WTC-associated elements in greater concentrations than found in autopsy tissues from less exposed controls. We also investigated whether post-9/11-onset respiratory symptoms assessed at the time of WTCHR enrollment were associated with WTC exposure as estimated by concentrations of elements in lung tissue using case-control analyses. Further research might validate elemental concentrations as biomarkers, i.e., quantitative measures of WTC exposure. Such exposure measures could be helpful to epidemiologic case-control and cohort studies by improving the accuracy of dose estimation, which is critical to dose–response analyses, which are often used in assessing whether exposure might be causally associated with observed diseases.

## 2. Materials and Methods

### 2.1. WTCHR Decedents

Between September 2003 and November 2004, the NYC Department of Health and Mental Hygiene (DoHMH) enrolled 71,437 individuals in the WTCHR [20,21]. WTCHR enrollees included (a) individuals who were present south of Chambers Street, lower Manhattan, on the morning of the disaster; (b) workers and volunteers involved in rescue, recovery, clean-up and other activities at the WTC site; (c) people with a primary place of residence south of Canal Street, lower Manhattan, on 11 September 2001; and (d) students and staff enrolled or employed on 11 September 2001 in schools (grades pre-kindergarten to 12) south of Canal Street. DoHMH staff invited individuals to enroll in the WTCHR if they were on various lists of eligible workers or residents, while other participants enrolled in response to advertising [20,21]. Enrollees completed a Wave One (W1) WTCHR enrollment questionnaire administered either via computer-assisted interview (95%) or face-to-face interview (5%) [21].

At enrollment in the WTCHR in 2003–2004, individuals responded to questions regarding the severity of exposure to WTC-associated dust and fumes, and the development of symptoms after the WTC disaster (see the WTCHR Wave 1 questionnaire, https://www1.nyc.gov/assets/911health/downloads/pdf/wtc/wtc-questionnaire.pdf accessed on 3 February 2014). Symptoms analyzed in the present study included wheezing, shortness of breath, cough and sinus problems [22]. Jordan, et al. [22] developed 2 scales of WTC exposure severity, including one for rescue and recovery exposure and one for non-rescue, non-recovery exposure. In each case, exposure was categorized as none, low, intermediate or high. Due to small numbers of subjects in some of these categories, we dichotomized rescue/recovery exposure into “none” vs. “high” (into which we combined “low”, “intermediate” and “high”). Non-rescue/recovery exposure was collapsed into 3 categories, “low”, “intermediate” and “high”.

The NYC OCME conducts autopsies of individuals who died in NYC when death is due to or suspected of being due to criminal violence, accident or suicide; when death suddenly occurs in an individual in apparent good health and unattended by a physician; when death occurs in a correctional facility; and when death occurs under suspicious or unusual circumstances, consistently with internationally accepted guidelines for autopsy conduct [23]. The Bureau of Vital Records, NYC DoHMH, carried out a record match that found 88 of the above-described WTCHR enrollees to have died during 2007–2011 and to have been autopsied by the OCME.

### 2.2. Community Controls

We initially identified 88 “community controls” from the roster of the autopsied cases that the OCME processed during the same time period as the WTCHR cases. We further limited community controls to individuals who were not enrolled in the WTCHR and were matched to the WTCHR decedents by age at death (in 5-year age groups from 15–19 to 85–89 years), sex, calendar quarter of death, race/ethnicity (White/non-Hispanic, Hispanic, Black/non-Hispanic, Asian and Pacific Islander) and education (≥some college vs. ≤high school or graduate equivalency degree). For 10 community controls, the stratified age-matching criterion had to be relaxed, in which case, the nearest-age control satisfying all other criteria was selected despite being in a different 5-year age stratum. Community controls, furthermore, were limited to individuals who resided at the time of death in areas of NYC that were in the 0–74th percentiles of exposure to WTC particulates in the 5 days after the disaster (as shown in a map on page 644 of [24]), i.e., individuals residing at the time of death anywhere in the Bronx; Manhattan, excluding southern Manhattan community districts numbers 101–106 (see NYC Community District maps at https://a816-health.nyc.gov/hdi/profiles/ accessed on 13 October 2023); Queens, excluding community districts 402, 404–406, 408 and 411 (areas east of the WTC site) and Rockaway zip codes 11694, 11695 and 11697; Staten Island, excluding zip codes 10301, 10304, 10305, 10306 and 10308; and Brooklyn, excluding all areas except community districts 05 and 16. No significant increase in respiratory symptoms was found in areas above the 74th percentile compared to those at or below during a 4-week period approximately 6 months 11 September 2001 [24].

Resource limits prevented analysis of the entire sample of 88 cases and matched community controls, so we selected 76 cases and 55 community controls at random from the original study for chemical and statistical analyses. The resultant groups were similar in the prior matching criteria. We, therefore, considered the study to be “frequency-matched” by the matching criteria described above.

### 2.3. Characteristics of WTCHR and Community Control Decedents

In addition to data in the WTCHR database, information on all subjects was available from death certificates and an electronic database maintained by the OCME. These data contributed causes of death, usual occupation and usual industry.

### 2.4. Autopsy Specimens

The OCME stores post-mortem macroscopic (gross stock tissue) tissue samples from all completed autopsies for at least 3 years. We chose to analyze tissues from individuals whose deaths occurred during 2007–2011. The standard procedure for OCME pathologists conducting autopsies was to obtain samples of all organ systems except for skin, hair and teeth. Standard anatomical locations for sampling within each organ were not specified. During that period, the OCME stored gross stock tissue specimens from each autopsy in one–three 1 L plastic jars, each containing 500 mL of 10% buffered formalin. Ultra-clean methods and materials were neither employed during autopsy nor during the extraction of samples for storage. Formalin used for the storage of tissue samples was not ultra-pure.

From the stored lung specimens, one of us (J.K.G.) excised, when possible (i.e., when doing so would not have consumed all of the remaining sample), a sample of “central lung” and a sample of “peripheral lung”, with these categorizations being based upon physical examination of the tissue specimen. Excisions were conducted using sterile plastic forceps and sterile stainless-steel scalpel blades. “Central” specimens were located toward the pulmonary hilum and contained medium or large cartilage-bearing bronchi and possibly peribronchial lymph nodes. “Peripheral” specimens were from the periphery of the lung and lacked medium or large bronchi or abutted the pleural surface of the lung. Some samples were considered “mixed” and could not be characterized as either peripheral or central lung. Samples for the present study were judged to be peripheral lung in 117 (89.3%), central lung in 11 (8.4%) and mixed central and peripheral in 3 (2.3%) cases. The distribution of sample loci did not differ significantly by group (WTCHR vs. community controls) (*p* = 0.3).

### 2.5. Laboratory Analyses

#### 2.5.1. Tissue-Drying Procedures and Inductively Coupled Plasma Mass Spectrometry (ICP-MS)

We desiccated lung tissue samples using a microwave method (Titan MPS; Perkin Elmer). The desiccated tissue was then homogenized with a Polytron homogenizer in ~0.5 mL of saline, digested in high-purity nitric acid (Fisher Optima grade) and analyzed using ICP-MS (NexION 350D).

We used ICP-MS, run in standard mode, to analyze metals in the dissolved, desiccated tissues, as this method can provide accurate measurements at concentrations as low as parts per trillion. Tissue concentrations of the measured elements were corrected by subtracting the mean concentration of each element observed in a series of blanks (containing the same non-ultra-pure formalin in which the WTCHR and community control samples had been stored) digested and run through the ICP-MS device on the same day as the specimens of interest. When this calculation resulted in negative values, we replaced the negative values with zero. When using log-transformed elemental concentrations in statistical analyses, we added 0.00001 to all values so that 0 s would not result in indeterminate values when log-transformed. We repeated all analyses by substituting values below each element’s limit of detection with the limit of detection divided by the square root of 2. We report the analyses replacing negative values with zero because the limits of detection, determined in our laboratory and based on small numbers of samples, were themselves imprecise.

#### 2.5.2. Quality Assurance and Quality Control

Standard reference specimens from the National Institute of Standards and Technology (NIST), including NIST 1577c bovine liver specimens and NIST 1566b oyster tissue, were subjected to the same procedures as the samples of interest in order to evaluate the reproducibility and recovery of the measured elements. During the course of laboratory analyses, we conducted 13 separate measurements of the concentrations of elements in these standards. Recovery percentages were calculated by dividing the median measured value of an element over the 13 replicates by the known concentration provided by the NIST. The “relative interquartile range” (RIQR) for each element was calculated as the 75th percentile (Q3) minus the 25th percentile (Q1) of the measured values divided by the median value and expressed as a percentage (%) of the median. We defined unreliable those elements for which the RIQR exceeded 20%.

### 2.6. Expected Values

A study of normal tissue obtained from lung biopsies of cancer cases in the United Kingdom [25] provided the expected elemental concentrations in lungs of individuals not exposed to the WTC disaster. In that study, 93% of individuals’ reason for biopsy was cancer, but the samples analyzed were excised from pathology-free lung areas. There were 54 subjects; the age range was 18–83 years (mean = 67.8 years); and 31 (57.4%) were male. We report median, and the 5th and 95th percentiles of the distributions of chemical concentrations in WTCHR decedents vs. community controls and among WTCHR decedents by exposure to the dust cloud for consistency with Morton, et al. [25]. Elsewhere, we report the 25th (Q1) and 75th (Q3) percentiles.

### 2.7. Prior Analyses

The data presented in this manuscript were obtained from new chemical analyses supplanting those previously reported by I-Hsin Lin and the present authors [26,27]. In the present analyses, we used a microwave digestion method instead of the previously employed hotplate method in order to reduce the chances of sample contamination by ambient particulates within the older fume hood. The present ICP-MS analyses were conducted with our new NYU NexION ICP-MS, rather than the Thermo Element magnetic-sector high-resolution ICP-MS device that we previously used at Lamont-Daugherty Earth Observatory, Columbia University, Palisades, NY, USA.

### 2.8. Statistical Methods

#### 2.8.1. Multiple Comparisons

We view our analyses as hypothesis-generating, aimed at identifying trace or major elements in lung tissue as possible biomarkers of exposure to the WTC disaster. Our primary analyses considered each of the measured elements individually. Some results may be statistically significant by chance alone due to the many statistical analyses we conducted. Despite this possibility, we chose not to adjust the study’s p-value for significance in order to increase our chances of bringing attention to possibly interesting associations. Following Rothman, we choose to depend on future research to confirm or refute our findings [28]. We used log-transformed elemental concentrations in regression analyses so as to reduce the influence of a small number of relatively high concentrations.

#### 2.8.2. One-Sided Statistical Tests

When contrasting chemical concentrations in the lung tissues of WTC-exposed individuals with those among community controls, we used one-sided statistical tests, because it would be uninteresting and counterintuitive to find elemental concentrations that decreased as exposure increased. For the same reason, we calculated one-sided confidence limits for regression coefficients representing the predicted increase in log-transformed elemental concentrations per unit increase in self-reported severity of WTC exposure. We employed separate exposure scores for rescue/recovery and non-rescue/recovery activities. If the confidence interval (CI) of positive regression values crossed into negative values, we truncated the CI at 0. If the estimate from the regression analysis was negative, we did not report the estimate, unless the upper limit of the 95% one-sided CI exceeded zero.

#### 2.8.3. Two-Sided Statistical Tests

In testing whether elemental concentrations were predictors of symptoms, we used two-sided statistical tests because pathological, symptom-related metabolic processes might have resulted in reductions in elemental concentrations among those with symptoms.

#### 2.8.4. Comparison of WTCHR and NYC Community Control Decedents

We used the chi-squared statistic or Fisher’s exact test to compare categorical variables between the WTCHR and community control groups. The distributions of elemental concentrations were non-normal, and some remained so after log transformation. We evaluated the statistical significance of differences in continuous variables with the non-parametric Wilcoxon rank sum test with statistical significance requiring a one-sided *p* < 0.05. The direction of difference from the rank sum test was deemed in the expected direction if the sum of Wilcoxon score statistics for an element in the WTCHR group exceeded the expected value under the null hypothesis.

For WTCHR decedents, we had access to self-reported WTC exposure, smoking history and respiratory symptoms, obtained at enrollment in the WTCHR, prior to death. We used these data to investigate, using Wilcoxon rank sum tests, whether lung concentrations of elements were positively correlated with WTC exposure, including exposure to the dust cloud (yes vs. no) and intensity of rescue/recovery and non-rescue/recovery exposure adjusted for age and smoking status. We also used the Wilcoxon test and logistic regression to investigate whether new-onset, post-9/11 symptoms of wheezing and shortness of breath were associated with lung tissue elemental concentrations.

### 2.9. Validity of Methods

To test the ability of the methods to detect retained contaminants from respiratory exposure, we examined the association of smoking history with chemical concentrations. In these analyses, we compared lung tissue concentrations in subjects that the OCME had classified as cigarette smokers to those in subjects that the OCME had not classified so. The OCME’s smoking classification of each autopsy subject was based on information from next of kin/family members, death scene investigation information, information contained in (available and reviewed) medical/clinical records and autopsy findings. Cd has been shown to accumulate in the lungs of cigarette smokers with chronic obstructive lung disease [29], so demonstration of an association of Cd with smoking would validate our methods.

## 3. Results

### 3.1. Reliability of Measurements of Chemical Concentrations

Good recovery percentages, 80–120%, were obtained for elements in the NIST 1577C bovine liver standard (Table 1) with the exception of Cr (recovery of 1628%), As (recovery of 141%), Sb (recovery of 32%) and Pb (recovery of 29%).

Good recovery percentages also were obtained for elements in the NIST 1566b oyster tissue standard (Table 2) with the exception of Sn (recovery of 57%), Sb (recovery of 61%) and Pb (recovery of 72%).

Ti was not included in either of the NIST standards, so we were unable to calculate recovery for this element. Thirteen replicate measurements of one subject’s lung specimen provided a median Ti concentration of 21.1 ppm and an RIQR of 50.7%, indicating poor reliability. Despite some elements having greater variability as according to elevated RIQRs and some elements (especially Cr) having poor recovery statistics, we included all measured elements in our analyses to ensure that potential insights were not overlooked.

### 3.2. Validity of Technique

Assuming that individuals not categorized as smokers by the OCME were non-smokers, the prevalence of smoking was 21.1% (16/76) in WTCHR decedents and 21.8% (12/55) in community controls (ns). The median Cd concentration in the lungs of the 28 smokers was 4.47 μg/g (95% IQR = 2.32, 5.85 μg/g) compared with 1.70 μg/g (95% IQR = 0.67, 3.30 μg/g) in the 108 non-smokers (*p* from Wilcoxon rank sum test < 0.0001). We thus considered our techniques validated, as they supported the known elevation of Cd concentration in lung tissue of smokers [25].

### 3.3. Study Subjects

The distributions of states and counties of residence differed between autopsied WTCHR decedents and the rest of the WTCHR population due to the selection criterion that autopsies had to have been performed by the NYC OCME (Table 3). Autopsied decedents also were older on 9/11 than other WTCHR participants, more likely to be male, non-White and less likely to be college-educated (Table 3).

### 3.4. WTCHR Decedents vs. Community Controls

As recorded on their death certificates, the WTCHR decedents (*n* = 76) and NYC community controls (*n* = 55) were of similar age (median = 54, IQR = 48, 62 years in WTCHR decedents vs. median =53, IQR = 46, 63 years among community controls; *p* = 0.9), gender (68% male vs. 74.5% male; *p* = 0.4) and education (high school graduate or graduate equivalency degree (GED) or less = 47% vs. 45.5%, *p* = 0.9). Due to the place-of-residence selection criterion for community controls, the distributions of boroughs of residence at the time of death differed between WTCHR decedents (32% Manhattan, 12% Bronx, 24% Brooklyn, 17% Queens, 7% Staten Island and 9% outside NYC) and community controls (31% Manhattan, 20% Bronx, 2% Brooklyn, 40% Queens and 7% Staten Island). More WTCHR decedents died in hospitals, emergency departments or outpatient clinics (38%) than community controls (29%), but the difference was not statistically significant (*p* = 0.3). Causes of death did not differ significantly between WTCHR decedents and community controls (*p* = 0.9). The most common causes of death in the two groups were heart disease (30% of WTCHR decedents and 24% of community controls) and injuries, including vehicular crashes, falls, violence and other causes (38% of WTCHR decedents and 42% of community controls).

### 3.5. Elemental Concentrations in WTCHR Decedents vs. Community Controls

Table 4 shows median, and 5th and 95th percentile concentrations of the 26 measured elements in lung specimens of WTCHR decedents and community controls, along with measurements of 20 of these elements reported by Morton, et al. [25]. The one-sided Wilcoxon rank sum test indicated that only the concentration of Fe in WTCHR decedents significantly exceeded that in community controls in the direction expected (one-sided *p* = 0.01). Fe continued to be the only element that differed significantly (*p* = 0.01) when these analyses were repeated by replacing values lower than the element-specific limits of detection with the limit of detection divided by the square root of 2, as opposed to being forced to zero. Fe concentrations among community controls were similar to those reported by Morton, et al. [25].

**Figure 1:**
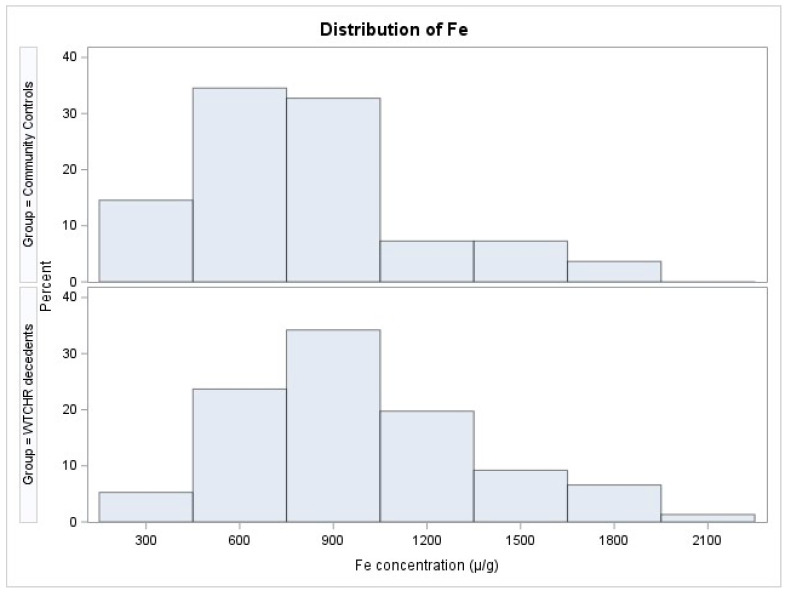
Lung tissue Fe concentrations of World Trade Center Health Registry (WTCHR) decedents and community controls. Fe concentration (μg/g).

The distributions of Fe in lung specimens of WTCHR decedents and community controls are shown in Figure 1, revealing a clear right shift in the WTCHR group’s distribution compared with that of the community controls.

We further analyzed elemental concentrations in lung tissue with regression analysis, employing log-transformed elemental concentrations as dependent variables. Rank-transformed values were rejected as dependent variables out of concern that ranks would have artificially magnified differences for elements in which many subjects had low or non-detectable concentrations. After adjustment for age and smoking status, these regression analyses confirmed that only Fe concentrations differed significantly between WTCHR decedents and community controls in the hypothesized direction (adjusted beta predicting log(Fe) concentration for membership in the WTCHR group compared with controls = 0.17, 95% one-tailed CI = 0.04, 0.30). Adding adjustment for year of death to the model did not alter this result.

### 3.6. Dust-Cloud Exposure among WTCHR Decedents

Among the 76 WTCHR decedents, 39 (51.3%) had reported being in the dust cloud. Non-parametric comparisons indicated significant elevations in lung tissue concentrations of Ti, Cr and Cd among those exposed to the dust cloud compared with those not exposed to the dust cloud (Table 5). Replacing values below the limits of detection with the limit of detection divided by the square root of 2 continued to show significant elevations in Cd, Cr and Ti. Multivariable regression analyses of log-transformed lung tissue concentrations found none of the measured elements to be significantly associated with dust-cloud exposure after adjustment for age and smoking status.

Regression analyses in which rescue/recovery and non-rescue/recovery exposure was considered after adjustment for age and smoking status showed the intensity of non-rescue/recovery exposure to be significantly and positively associated with log(Mo) concentrations (beta = 0.24, 95% one-tailed CI = 0.11, 0.37).

### 3.7. Chemical Concentrations and Death Certificate Notations of Usual Occupation and Industry

In a post hoc inspection of the data, we sorted subjects by Fe concentration and listed the usual occupations and industries as recorded on the subjects’ death certificates. Seven individuals in the combined group of WTCHR decedents and controls had Department of Sanitation listed in these fields, and these subjects’ Fe concentrations fell in the top 43 percentiles of the Fe concentration distribution. All seven were WTCHR decedents; they had a median Fe concentration of 1281 (IQR = 1049, 1893 µg/g), which was similar to the values observed in two NYC Police Department employees (values of 1246 and 1441; median = 1343 µg/g), higher than that observed among five Fire Department of New York employees (median = 925; IQR = 732, 1224 µg/g) and substantially higher than that observed among all subjects whose death certificates did not mention the Sanitation, Fire or Police Departments in the “usual occupation” or “usual industry” death-certificate fields (*n* = 117, median = 810; IQR = 624, 1047 µg/g).

### 3.8. Symptoms and Lung Concentrations of Measured Elements among WTCHR Decedents

The Wilcoxon signed rank test indicated significantly increased lung tissue concentrations among WTCHR decedents for As among those having reported new-onset wheezing; Co and As among those having reported new-onset shortness of breath; As among those having reported new-onset cough; and Mn, Co and As among those having reported new-onset sinus problems. Significantly reduced lung tissue concentrations in WTCHR decedents with new-onset respiratory symptoms compared with those without were found for Fe among those having reported new-onset wheezing or new-onset sinus problems and for Se among those having reported new-onset shortness of breath. Elemental lung tissue concentrations with at least one significant odds ratio after adjustment for age and smoking status are shown in Table 6.

## 4. Discussion

We found significantly increased Fe concentrations in lung tissue of WTCHR decedents, consistently with steel being one of the primary components of WTC dust [12]. Fe-containing particles previously have been found in biopsy lung samples from WTC-exposed individuals [16]. Mo concentrations were significantly associated with non-rescue/recovery exposure. While Ti, Cd and Cr concentrations were elevated among WTCHR decedents who had reported having had dust-cloud exposure, these associations were not statistically significant after adjustment for age and smoking status. Inadequate precision in the adjustment for age and smoking status might in part explain the lack of significance of associations between these elements and WTC dust-cloud exposure in multivariable analyses. Cr and Fe are found in steel, which is a common component of WTC exposure. However, our measurements of concentrations in NIST standards for Cr indicated substantial variability. Therefore, the small sample size and low abundance (compared with Fe) might have contributed to the loss of statistically significant associations with WTC dust-cloud exposure status after adjustment for covariates in multivariable regression analyses.

We had expected to find increased concentrations among WTCHR decedents of less common, more exotic elements, such as those found in electronic components, furniture, or paints. Instead, we found elevated concentrations of a major element, Fe, and a minor, trace element, Mo, which was associated with exposure to WTC contaminants through non-rescue/recovery activities. The Fe finding was further supported by elevated Fe concentrations in lung tissues of individuals identified, on death certificates, as NYC Sanitation or Police Department employees. Both groups are known to have experienced severe WTC exposure [5,31]. As Mo was used in the manufacture of some of the steel components of the WTC [32], the Mo finding, coupled with the Fe finding, suggests the presence of steel shards from the collapsed WTC towers’ infrastructure in lung tissues. Inadequate sample size might be another major reason for the lack of statistically significant association of Cr with dust-cloud exposure after adjustment for age and smoking status.

Failure to find differences in minor elements, such as Sb, might be explained by several factors. First, the measurements of some elements were highly variable according to our quality control evaluations of known concentrations in NIST standards. Second, the WTC dust clouds were probably not well mixed, so exposure to less common materials in particular might have varied greatly among individuals depending on chance and distance from the towers. Third, WTC exposure may have occurred among some of the community controls. While inclusion criteria required that community controls were not enrolled in the WTCHR and resided, at the time of death (in 2007–2011), in areas of NYC that were less impacted by WTC dust, it is possible that some may have resided in and/or traveled on 9/11 to locations that would have qualified them for WTCHR enrollment and may have subjected them to substantial WTC exposure.

The validity of our methods was supported by our finding of an association of smoking with the concentration of Cd in lung tissue. The smoking status in these analyses was taken from OCME ascertainments based on clinical history, the querying of family and pathology at death. Some smoking history may have been incorrect, especially in failing to identify smoking many years before death. Nonetheless, our methods were validated by the replication of the previously reported association of smoking with Cd lung concentrations [25].

Our methods also were supported by the testing of NIST samples with published concentrations of most of the 26 elements we studied. We provide our observations of Mo concentration in uncertified reference material NIST 1566b and hope others find this measurement useful.

Future studies of possible biomarkers of WTC exposure might consider measuring in vivo bone concentrations of Fe and Mo using X-ray fluorescence, assuming that the retained particulates in lung tissue leach over time into the circulatory system and then sequester in bone. Another option might be to use T2* magnetic resonance imaging (MRI), which has been used to measure Fe in heart, liver and cancer tissues [33,34,35]. MRI would be costly, but in small studies, it might be used to obviate radiation exposure associated with X-ray fluorescence among individuals who already have substantial health concerns.

The negative associations we observed between Fe and respiratory symptoms is supported by the known reduction in Fe lung concentrations among individuals with asthma [36]. To mitigate both the oxidative stress presented by Fe and other toxic metals, and their potential for tissue injury, lung cells dynamically alter Fe import and export pathways to control redox balance, Fe storage and utilization [37]. Disruption of Fe homeostasis has been shown to be associated with infection, acute lung injury and chronic lung disease [37]. Higher levels of Fe in lung tissues of WTCHR decedents without respiratory symptoms compared with those with such symptoms may in some small way reflect positive impacts of increased Fe concentrations on lung injury and disease.

Our findings could have been influenced by physiological mechanisms and varying periods between exposure to the World Trade Center disaster and autopsy. We do not believe such mechanisms could have accounted for our findings, as adjustment for year of death did not alter the findings substantially. We also adjusted for age to account for differing lengths of life and thus lifetime accumulation of Fe. Discussion of the biokinetics of Fe, including Fe in lung tissues, can be found in Ali, et al. [36]. It is possible that differences in commuting patterns could have accounted for some of the differences we observed in Fe exposure, as high exposure to Fe-containing particles has been reported in the New York City subway system [38]. Unfortunately, data on subway usage by WTCHR decedents or community controls were not available.

We found increased odds ratios for respiratory symptoms associated with As, Co and Co. All four symptoms that we investigated (new-onset post-9/11 wheezing, shortness of breath, cough and sinus problems) had significantly increased odds ratios associated with greater lung concentrations of As. As is known to be associated with respiratory symptoms [39] and was found in WTC dust [12].

There are numerous potential factors that may have adversely affected our effort to relate chemical concentrations in lung tissue at autopsy to WTC exposure. Changes in chemical concentrations, for example, may have occurred between the time of death and the time of receipt of the corpses by the OCME due to tissue decomposition. Alterations in chemical concentrations could have occurred from contamination during removal of samples by the present investigators from the OCME storage jars, excision of subsamples, temporary storage and shipping to our chemistry laboratory, as well as during digestion, desiccation and ICP-MS. While samples had been stored by the OCME in non-ultra-pure formalin, the same was used in blanks that provided the background levels that were subtracted from the measurements of the samples of interest. We, therefore, do not believe that our results could reflect metals in the formalin.

Our current findings differ substantially from the findings presented by Lin in her PhD thesis [26,27]. Whereas Lin found that Ag was significantly higher and that U was significantly lower among autopsied WTCHR participants compared with NYC community controls, we found that Fe was higher in the WTCHR decedents and that Mo was elevated among WTCHR decedents with non-rescue/recovery exposure. Lin found that Al was higher in central lung tissues of rescue/recovery workers and that Fe was higher among WTCHR participants with non-rescue/recovery exposure. In addition to possible contamination of some of the samples studied by Lin (a problem we believe has been reduced or eliminated using the current methods), differences between the two studies may be due to differences in the subject population, the specific samples entered into the analyses and the statistical methods employed.

We did not adjust our statistical evaluations for multiple comparisons despite the numerous analyses contained in this report covering 26 elements, several self-reported exposure measures, and 3 symptoms. We did not adjust the present study’s *p*-value cut-point for significance to reflect the many comparisons, because we view this study as hypothesis-generating. As such, our findings will be best validated or refuted through replication in other samples and study designs [28]. Adjustment for multiple comparisons would have reduced our chances of identifying potentially interesting biomarkers for future investigation.

A strength of our study was the demonstrated ability to measure with good accuracy and reliability the concentrations of most of the elements we studied in NIST biological standards with known concentrations. A weakness, however, was that our measurements of concentrations in NIST standards for Cr and several other elements indicated substantial inaccuracy and variability. The certified concentrations were sometimes much lower than the concentrations we measured in the autopsy tissues. We, nonetheless, analyzed and reported these elemental concentrations because the same measurement methods were applied to the two groups. There was no reason to believe that excursions in laboratory measurements above or below true values would occur more in one exposure group than another. Our primary finding of an increased concentration of Fe in the WTCHR group compared with the community controls was strengthened by the similarity of the Fe concentration we found among community controls (median = 759 μg/g) to that found by a study conducted in the United Kingdom of human lung concentrations in which the median Fe concentration among 54 donors was 746 μg/g [25].

Histologic evaluations of lung tissues are not routinely conducted by the OCME. Future studies might compare the prevalence of histologically identified lung conditions and their association or non-association with lung concentrations of Fe in particular and possibly the other elements, including Mo, Ti, Cr and Cd, for which there were suggestions of utility as biomarkers of WTC exposure.

The importance of establishing objective measures of WTC exposure for ongoing research of WTC health effects cannot be overstated. Dose–response relationships are critical to establishing whether different diseases observed among WTC survivors are in fact causally related to WTC exposure. Accurate exposure characterization, classification of relative exposure intensities and identification of unrecognized exposure among control samples are all key to causal inference. We hope that our tentative identification of Fe and to a lesser extent Mo, Ti, Cr and Cd as possible biomarkers of WTC exposure might be replicated in the future and thus permit these elements to serve as biomarkers of WTC exposure. It is possible that these elements could improve epidemiologic studies of WTC health effects and clarify the role of WTC exposure in the etiology of different diseases being observed in the WTCHR population, especially through case-control studies.

## 5. Conclusions

In sum, the data support our hypothesis that retained elements in the lung might suggest biomarkers for further study as potential indicators of 9/11-related exposure. Our analyses specifically suggested that Fe was increased among WTC-exposed decedents compared with community controls. Within the WTCHR group, Mo, an element known to have been in WTC dust [12], was significantly elevated among individuals with higher self-reported exposure.

Inaccurate estimation of exposure can lead to misclassification, potentially biasing epidemiologic associations toward the null. Imprecise exposure estimates also can make dose–response relationships difficult to discern. The present study suggests a response to these concerns, i.e., the investigation of trace and major elements in the lung, blood, serum, urine, bone and nails from WTC survivors might reveal long-term biomarkers of WTC exposure.

The overall findings suggest that major and trace elements from the WTC disaster remained in human lung tissues 6–10 years after the event and that further work to explore the potential utility of these elements as biomarkers of exposure among the living is warranted. When used in concert with self-reports, biomarkers of WTC exposure could improve the accuracy of epidemiologic investigations of WTC health effects. Biomarkers of exposure also may lead to improved medical interventions if diseases initiated or promoted by WTC exposure require different treatments from the same diseases initiated or promoted by other factors.

## Figures and Tables

**Table 1:** Chemical concentration measurements and chemical concentrations from 13 replicate measurements of NIST 1577c bovine liver standard specimens. All concentrations are in parts per million.

Element	NIST 1577c Conc. (ppm)	Q1 (ppm)	Median (ppm)	Q3 (ppm)	Recovery (Median/ NIST Conc.)	RIQR (IQR/ Median)
Beryllium (Be)		0 ^†^	0 ^†^	0 ^†^		
Sodium (Na)	2033	1802	1817	1883	89%	4%
Magnesium (Mg)	620	541	554	582	89%	8%
Aluminum (Al)		0.325	0.666	1.47		173%
Potassium (K)	10,230	9640	9900	11,100	97%	15%
Calcium (Ca)	131	129	141	147	108%	13%
Titanium (Ti)		16.5	21.1	27.2		51%
Vanadium (V)	0.00817	0.0054	0.0090	0.0136	110%	92%
Chromium (Cr)	0.053	0.33	0.86	0.92	1628%	69%
Manganese (Mn)	10.46	9.14	9.52	10.6	91%	15%
Iron (Fe)	197.94	169	178	198	90%	16%
Cobalt (Co)	0.300	0.272	0.302	0.308	101%	12%
Nickel (Ni)	0.0445	0 ^†^	0.053	0.067	119%	127%
Copper (Cu)	275.2	243	252	277	91%	14%
Zinc (Zn)	181.1	165	181	194	100%	16%
Arsenic (As)	0.0196	0.0085	0.028	0.035	141%	96%
Selenium (Se)	2.031	1.82	2.01	2.39	99%	28%
Strontium (Sr)	0.0953	0.065	0.080	0.094	84%	36%
Molybdenum (Mo)	3.30	3.01	3.16	3.45	96%	14%
Silver (Ag)	0.0059	0.00003	0.0047	0.0096	80%	202%
Cadmium (Cd)	0.097	0.082	0.098	0.104	101%	22%
Tin (Sn)		0 ^†^	0.00179	0.00829		462%
Antimony (Sb)	0.00313	0 ^†^	0.0010	0.0021	32%	211%
Barium (Ba)		0.0304	0.0377	0.0427		32%
Thalium (Tl)		0 ^†^	0 ^†^	0.00096		
Lead (Pb)	0.0628	0 ^†^	0.018	0.064	29%	351%

^†^ indicates that the 25th percentile had all replaced values, which occurred when daily field blanks were >sample results, in which case negative values were replaced with 0 s.

**Table 2:** Chemical concentrations from 13 replicates measurements of NIST 1566b oyster tissue standard specimens, stated concentrations in the NIST standard, recovery percentages and relative interquartile ranges.

Element	NIST 1566b Conc. (ppm)	Q1 (ppm)	Median (ppm)	Q3 (ppm)	Recovery (Median/ NIST Conc.)	RIQR (IQR/ Median)
Ba		0.00000	0.00213	0.00575		270%
Na	3297	2980	3060	3140	93%	5%
Mg	1085	995	1015	1050	94%	6%
Al	197.2	158	172	183	87%	15%
K	6520	6143	6220	6630	95%	8%
Ca	838	880	902	963	108%	9%
Ti		19.7	22.2	27.0		33%
V	0.577	0.521	0.542	0.586	94%	12%
Cr	0.950 **	0.840	0.916	1.14	96%	32%
Mn	18.5	17.2	18.2	19.0	98%	10%
Fe	205.8	186	196	203	95%	9%
Co	0.371	0.333	0.360	0.366	97%	9%
Ni	1.04	0.905	0.933	0.995	90%	10%
Cu	71.6	65.0	66.2	68.0	92%	4%s
Zn	1424	1310	1380	1460	97%	10%
As	7.65	6.842	7.06	7.58	92%	10%
Se	2.06	2.287	2.42	2.82	117%	22%
Sr	6.8	5.9	6.3	6.5	92%	9%
Mo		0.144	0.156	0.164		13%
Ag	0.666	0.585	0.625	0.647	94%	10%
Cd	2.48	2.45	2.50	2.51	101%	2%
Sn	0.031	0.0074	0.018	0.021	57%	79%
Sb	0.011	0 ^†^	0.0067	0.0103	61%	155%
Ba	8.6	7.71	8.12	8.22	94%	6%
Tl		0 ^†^	0.00028	0.00423		1509%
Pb	0.308	0 ^†^	0.22	0.30	72%	135%

** Concentration reported by Souza, et al. [30]. ^†^ indicates that the 25th percentile had all replaced values, which occurred when daily field blanks were > sample results, in which case negative values were replaced with 0 s.

**Table 3:** Demographic characteristics of the autopsied vs. all other enrollees (from data provided at enrollment into the World Trade Center Health Registry).

	Autopsied Enrollees	All Other Registry Enrollees *	*p*-Value **
	(*n* = 76)	(*n* = 70,494)	
State of Residence on 11 September 2001			
New York	75 (98.7%)	56,985 (80.9%)	<0.01
New Jersey	1 (1.3%)	7389 (10.5%)	
Other	0 (0.0%)	6044 (8.6%)	
Place of Residence on 11 September 2001			
Manhattan	28 (39.4%)	21,183 (43.7%)	0.21
Bronx	9 (12.7%)	3347 (6.9%)	
Brooklyn	18 (25.3%)	9716 (20.0%)	
Queens	11 (15.5%)	10,063 (20.8%)	
Staten Island	5 (7.0%)	4194 (8.6%)	
Age on 11 September (median, IQR)	(46; 54 − 39 = 15)	(40; 49 − 32 = 17)	0.02
0–17	1 (1.3%)	3148 (4.4%)	
18–24	4 (5.3%)	4353 (6.2%)	
25–44	29(38.2%)	36,731 (52.3%)	
45–64	37(48.7%)	23,556 (33.5%)	
65+	5 (6.6%)	2505 (3.6%)	
Gender			
Male	52 (68.4%)	42,323 (60.1%)	0.14
Female	24 (31.6%)	28,095 (39.9%)	
Race/ethnicity			
White	37 (48.7%)	44,304 (62.9%)	<0.01
Black	19 (25%)	8393 (11.9%)	
Hispanic	9 (11.8%)	9471 (13.5%)	
Asian	6 (7.9%)	5232 (7.4%)	
Multiracial/Other	5 (6.6%)	3018 (4.3%)	
Education—Highest Year of School completed			
Below college	28 (36.8%)	18,922 (27.3%)	0.06
College and above	48 (63.2%)	50,313 (72.7%)	

* Excluding the 76 autopsied subjects in this study and 930 WTCHR enrollees who withdrew from the Registry by 11 September 2015. Column totals may not add up to 70,494 due to missing values. ** *p*-Values are for chi-squared or Fisher’s exact tests.

**Table 4:** Elemental concentrations (µg/g) in lung tissues from WTCHR decedents and community controls, and concentrations measured in biopsy lung biopsy specimens reported by Morton, et al. [25].

	Reported by Morton, et al. (*n* = 54)		Community Controls (*n* = 55)			WTCHR Decedents (*n* = 76)			Sum of WTCHR Wilcoxon Scores ^†^	*p*-Value *
Element	Median	95th Percentile	Median	5th Percentile	95th Percentile	Median	5th Percentile	95th Percentile		
Be	0.00	0.01	0	0	0.0485	0	0	0.0143	4863	1
Na			16,754	10,233	25,501	16,253	10,108	28,264	4980	1
Mg			449	355	719	444	221	706	4823.5	1
Al	14.3	116	25.0	5.00	201	18.6	6.65	96.8	4754.5	1
K			7750	3540	12,137	7437	2828	12,960	4973	1
Ca			1533	459	3216	1228	359	2836	4668	1
Ti	1.59	15.7	0.134	0.018	61.3	0.212	0.0295	40.6	5250	0.14
V	4.86	22.1	0.258	0.000	0.569	0.200	0.000	0.567	4799	1
Cr	0.48	5.18	1.3	0.2	2.6	1.3	0.2	2.01	5018	0.50
Mn	0.621	2.01	0.893	0.300	2.60	0.743	0.216	2.88	4789	1
Fe	746	1692	759	316	1594	945	326	1770	5526	0.01
Co	0.11	0.42	0.0819	0.000	0.200	0.060	0.000	0.164	4739	1
Ni	0.221	1.13	0.318	0.0319	1.22	0.288	0.000	0.763	4726	1
Cu	6.02	10.51	9.5	5.82	15.6	9.2	5.5	16.7	5059	0.4
Zn	49.44	82.9	85.9	46.9	135	85.5	45.6	166	5030	0.5
As		0.168	0.00914	0.000	0.161	0.013	0	0.1	5059	0.4
Se	0.89	2.37	1.012	0.200	6.900	0.967	0	5.8	4752	1
Sr	0.32	0.83	0.7	0.283	1.33	0.600	0.246	1.8	4698	1
Mo	0.080	0.522	0.222	0.100	0.569	0.226	0.081	0.6	4984	1
Ag			0.00	0.00	0.02	0.00	0.00	0.02	4955	1
Cd	0.27	3.43	2.80	0.10	8.30	1.67	0.11	17.0	4726	1
Sn	1.01	6.66	0.400	0.0796	2.88	0.400	0.100	2.500	5003	1
Sb	0.03	0.12	0.051	0.000	0.453	0.057	0.000	0.200	5250	0.14
Ba	0.22	2.58	0.222	0.069	1.639	0.200	0.044	0.900	4879	1
Tl	0.0005	0.003	0.000	0.000	0.0461	0.000	0.000	0.00849	4991	1
Pb	0.08	0.23	0.078	0.000	0.900	0.100	0.000	1.536	5182	0.21

* One-tailed Wilcoxon rank sum test *p*-value. ^†^ Under the null hypothesis, the sum of squares for the WTCHR decedents for all elements = 5016.

**Table 5:** Elemental concentrations (µg/g) in lung tissues from WTCHR decedents and community controls, and 5th and 95th percentiles among WTCHR decedents by exposure to the WTC dust cloud. Elements with significant differences between WTCHR decedents and community controls are gray-shaded. Exposure to the WTC dust cloud on 11 September 2001.

	Dust-Cloud-Exposed (*n* = 39)			Dust-Cloud-Unexposed (*n* = 37)			One-Sided *p*-Value *
Element	Median	5th Percentile	95th Percentile	Median	5th Percentile	95th Percentile	
Be	0	0	0.0282	0	0	0.0082	0.4
Na	15,777	10,108	30,545	16,383	9940	28,264	1
Mg	439	215	689	453	221	741	0.4
Al	17.7	5.9	148	19.7	7.82	92.7	1
K	7944	2828	12,960	7378	2151	13,838	0.15
Ca	1249	359	2836	1107	348	2894	0.3
Ti	1.70	0.0224	49.2	0.122	0.0224	36.1	0.05
V	0.20	0.024	0.582	0.226	0	0.560	0.5
Cr	1.40	0.080	2.24	1.28	0.20	1.83	0.02
Mn	0.80	0.27	2.30	0.70	0.20	4.65	0.2
Fe	865	320	1657	1032	326	1928	1.0
Co	0.078	0	0.2	0.050	0	0.141	0.2
Ni	0.25	0.15	0.70	0.318	0	0.985	1
Cu	9.41	4.05	16.6	8.70	5.59	17.4	0.2
Zn	85.5	50.1	155.4	85.2	44.3	204	0.4
As	0.0103	0	0.20	0.018	0	0.10	0.5
Se	0.9333	0	1.68	0.978	0	8.30	0.5
Sr	0.60	0.25	1.78	0.60	0.24	1.82	1
Mo	0.26	0.08	0.55	0.22	0.063	0.747	1
Ag	0	0	0.02	0	0	0.021	1
Cd	2.0	0.10	19.6	1.57	0.20	7.70	0.05
Sn	0.50	0.10	2.50	0.30	0.062	2.30	0.15
Sb	0.057	0	0.394	0.057	0	0.20	0.4
Ba	0.20	0	0.80	0.2	0.081	0.92	0.5
Tl	0	0	0.0178	0	0	0.0038	0.4
Pb	0.10	0	1.64	0.1	0	1.54	1

* One-tailed Wilcoxon rank sum test *p*-value. Under the null hypothesis, the sum of squares for the WTCHR decedents for all elements = 5016.

**Table 6:** Odds ratios and 95% confidence intervals adjusted for age and smoking status for symptoms of new-onset, post-9/11 shortness of breath, cough and sinus problems, limited to elements with one or more significant odds ratios associated with log-transformed elemental lung tissue concentrations.

	Wheezing			Cough			Shortness of Breath			Sinus Problems		
Variable	OR	95% CI	*p*- Value	OR	95% CI	*p*- Value	OR	95% CI	*p*- Value	OR	95% CI	*p*- Value
Mn	0.94	0.48, 1.85	0.9	0.97	0.50, 1.90	0.9	1.03	0.53, 2.0	0.9	2.39	1.12, 5.09	0.02
Fe	0.27	0.08, 0.98	0.05	0.57	0.19, 1.69	0.3	0.53	0.17, 1.60	0.7	0.28	0.09, 0.91	0.03
Co	1.10	0.95, 1.27	0.19	1.05	0.92, 1.21	0.5	1.25	1.06, 1.48	0.007	1.22	1.04, 1.43	0.01
As	1.19	1.04, 1.36	0.01	1.15	1.01, 1.30	0.03	1.28	1.11, 1.48	0.007	1.17	1.03, 1.32	0.02

## Data Availability

Requests for access to data can be addressed to Dr. Mark Farfel, NYC DoHMH (mfarfel@health.nyc.gov). World Trade Center Health Registry data may be made available following review of applications to the Registry by external researchers. Mortality data may be requested from the NYC DoHMH Bureau of Vital Statistics.
